# Cultivable Alginate Lyase-Excreting Bacteria Associated with the Arctic Brown Alga *Laminaria*

**DOI:** 10.3390/md10112481

**Published:** 2012-11-06

**Authors:** Sheng Dong, Jie Yang, Xi-Ying Zhang, Mei Shi, Xiao-Yan Song, Xiu-Lan Chen, Yu-Zhong Zhang

**Affiliations:** State Key Lab of Microbial Technology, Marine Biotechnology Research Center, Shandong University, Jinan 250100, China; Email: Ds518@126.com (S.D.); 645670894@qq.com (J.Y.); zhangxiying@sdu.edu.cn (X.-Y.Z.); clubstone@sdu.edu.cn (M.S.); xiaoyansong@sdu.edu.cn (X.-Y.S.); zhangyz@sdu.edu.cn (Y.-Z.Z.)

**Keywords:** alginate lyase-excreting bacteria, psychrophilic, Arctic, *Laminaria*, diversity

## Abstract

Although some alginate lyases have been isolated from marine bacteria, alginate lyases-excreting bacteria from the Arctic alga have not yet been investigated. Here, the diversity of the bacteria associated with the brown alga *Laminaria* from the Arctic Ocean was investigated for the first time. Sixty five strains belonging to nine genera were recovered from six *Laminaria* samples, in which *Psychrobacter* (33/65), *Psychromonas* (10/65) and *Polaribacter* (8/65) were the predominant groups. Moreover, 21 alginate lyase-excreting strains were further screened from these *Laminaria*-associated bacteria. These alginate lyase-excreting strains belong to five genera. *Psychromonas* (8/21), *Psedoalteromonas* (6/21) and *Polaribacter* (4/21) are the predominant genera, and *Psychrobacter*, *Winogradskyella*, *Psychromonas* and *Polaribacter* were first found to produce alginate lyases. The optimal temperatures for the growth and algiante lyase production of many strains were as low as 10–20 °C, indicating that they are psychrophilic bacteria. The alginate lyases produced by 11 strains showed the highest activity at 20–30 °C, indicating that these enzymes are cold-adapted enzymes. Some strians showed high levels of extracellular alginate lyase activity around 200 U/mL. These results suggest that these algiante lyase-excreting bacteria from the Arctic alga are good materials for studying bacterial cold-adapted alginate lyases.

## 1. Introduction

Alginate is a liner unbranched copolymer of α-L-guluronate (G) and its C5 epimer, β-D-mannuronate (M), arranged in block structures such as homopolymeric G block, M block, alternating MG (GM) block, and heteropolymeric MG (GM) block [1]. Its unique physical properties to form gels enable it to be used widely as stabilizer, viscosifier, and gelling agent [2,3]. Alginate lyases degrade alginate by a β-elimination mechanism that generate a product containing 4-deoxy-L-*erythro*-hex-4-enopyranosyluronic acid as the non-reducing terminal moiety [4]. The non-reducing end groups of the products generate an increasing absorbance at 235 nm, which can be used to monitor real-time alginate lyase activity [5,6]. Alginate lyases are classified into three groups by their substrate specificity: the first type is specific toward G block (EC4.2.2.11), the second type is specific toward M block (EC4.2.2.3), and the third type is bifunctional for G and M block. Furthermore, alginate lyases are also grouped into three classes by their molecular masses: 20–35 kDa class, ~40 kDa class, and ~60 kDa class [7]. Based on their primary structures, alginate lyases are classified into seven families (PL5, 6, 7, 14, 15, 17 and 18) [8].

Alginate lyases are reported from various resources, including marine algae, marine mollusks, fungi, bacteria, bacteriophages, and viruses [9]. Because alginate is of great abundance as part of the cell wall and intracellular material in brown alga, some alginate lyase-excreting strains are separated from brown alga, such as *Pseudoalteromonas elyakoii* IAM 14594 from the spot-wounded fronds of *Laminaria japonica* [10] and *Pseudoalteromonas* sp. SM0524 from rotten kelp [11]. However, study on the diversity of brown alga-associated bacteria has been rather less extensive. Lee *et al.* (2006) isolated 66 strains from Korean brown alga *Undaria pinnatifida* [12], which represents the best investigation on the diversity of alga-associated bacteria to our knowledge. Moreover, the diversity of the alginate lyase-excreting bacteria associated with brown algae has not been reported.

The Arctic Ocean is the most extreme ocean regarding the seasonality of light and its year-round existing ice cover. A large number of unique life forms are highly adapted to the extreme environment of the Arctic Ocean in their ecology and physiology. Brown alga growing in the intertidal zone of the Arctic Ocean is a potential resource for the discovery of new bacteria producing novel alginate lyases, which, however, have never been investigated. In this paper, the diversity of cultivable bacteria associated with the brown alga *Laminaria* collected from the Arctic Ocean was investigated, and the alginate lyase-excreting bacteria were isolated from these samples and identified. Moreover, the alginate lyase production of these bacteria was detected, and the optimal temperatures for bacterial growth, alginate lyase production and the activity of alginate lyase from each strain were measured. The results are beneficial for exploring novel alginate lyase from the Arctic Ocean.

## 2. Materials and Method

### 2.1. Materials

Sodium alginate (~250 cps) from brown algae was purchased from sigma (USA). The plasmid pMD-18-T (Takara) and *Escherichia coli* DH5α were used for cloning and sequencing of 16S rRNA genes. The clones were grown on LB agar or liquid medium containing 100 μg/mL of ampicillin at 37 °C. Algal samples were collected from six locations in the intertidal zone of the Kings Bay in Ny-Ålesund, Svaldbard during the Chinese Arctic Yellow River Station Expedition in July, 2011. The numbers and locations of the six samples were shown in Table 1. The algal samples were stored in airtight sterile plastic tube at 4 °C until returning to the laboratory. The six algal samples were all identified as *Laminaria* sp. 

**Table 1:** The locations of sampling stations.

Station	Location	
0	78°55′3.0″N	11°58′14.0″E
1	78°55′19.7″N	11°56′53.8″E
2	78°55′17.0″N	11°57′0.2″E
3	78°55′3.8″N	11°58′11.6″E
B	78°55′20.5″N	11°54′49.7″E
C	78°55′3.8″N	11°56′53.8″E

### 2.2. Enrichment Cultivation and Isolation of the Bacteria Associated with the Arctic Brown Alga *Laminaria*

Each *Laminaria* sample was cut into small pieces using sterile scissors. One gram of every sample was put in 100 mL of the enrichment medium containing marine broth 2216 (Difco) plus 1% sodium alginate. After incubated at 15 °C, 200 rpm for 8 h, the culture broths were serially tenfold diluted to 10^−^^6^ dilution with sterile artificial sea water. Aliquots of 100 μL diluted samples (10^−^^2^–10^−^^6^ dilution) were spread on screening plates with a medium composed of 0.1% yeast extract, 0.5% tryptone, 1% sodium alginate, 1.5% agar powder and artificial seawater (pH 7.0). The plates were then incubated at 15 °C for a proper time to form detectable colonies. Morphologically different colonies were selected and further purified by repeatedly streaking on the same medium. The purified strains were stored in 10% glycerol at −80 °C.

### 2.3. Screening of Alginate Lyase-Excreting Bacteria

The purified isolates were, respectively, streaked on the plate with a medium containing 0.1% yeast extract, 0.5% tryptone, 0.4% sodium alginate, 1.5% agar powder and artificial seawater (pH 7.0). After being incubated at 15 °C for 4 days, the plates were stained by flooding with 10% (weight/volume) cetylpyridiniun chloride. If a strain excreted alginate lyase into the medium, a clear halo of depolymerization around the strain colony on a white background could be observed. Therefore, the isolates with a clear halo were selected as alginate lyase-excreting bacteria. 

### 2.4. Amplification, Cloning, and Sequencing of the 16S rRNA Gene

The genomic DNA of each isolate was extracted by using the BIOTEKE genomic DNA purification kit. The 16S rRNA genes were amplified from the genomic DNA by PCR with two primers (1492r: 5′-GGTTACCTTGTTACGACTT-3′, 27f: 5′-AGAGTTTGATCCTGGCTCAG-3′) [13]. The amplified DNA fragments of the 16S rRNA genes were ligated into pMD-18-T cloning vectors (Takara) and sequenced by Biosune Inc. (Shanghai, China). Sequence alignment was performed using CLUSTAL X (v 1.83). Neighbor-joining trees were constructed using MEGA version 3.1 with neighbor-joining method and Kimura two parameter model [14].

### 2.5. Assay for Alginate Lyase Activity

Alginate lyase activity was quantitatively determined by measuring the amount of reducing sugars released from alginate by using the Somogyi-Nelson method [15]. Bacterial culture was centrifuged at 12,000× *g*, 4 °C for 12 min. The supernatant was properly diluted with 20 mM phosphate buffer (pH 7.0) if needed. Then, 20 μL diluted culture supernatant was mixed with a 180 μL solution composed of 20 mM phosphate buffer (pH 7.0) and 0.5% sodium alginate. The mixture was incubated at an indicated temperature for 30 min. After incubation, 200 μL Nelson copper reagent was added, and then the mixture was bathed in boiling water for 15 min, followed by a fast cool in ice bath. Then 100 μL arsenomolybdate was added. Enzyme activity was determined by monitoring the absorbance increase at 680 nm. A standard curve was prepared using mannose at concentrations ranging from 0.1 to 1 mM. One unit of enzyme activity was defined as the amount of protein that generates 1 nmol reducing sugars per min. A blank control was set by mixing Nelson copper reagent and the substrate solution followed by an addition of culture supernatant to inactiviate the alginate lyase activity before incubation. 

### 2.6. Analysis of the Optimal Temperatures for the Growth and Alginate Lyase Production of the Isolates and for the Activity of Alginate Lyase from Each Strain

The alginate lyase-excreting bacteria were cultivated in the liquid screening medium at series temperatures, from 10 °C to 35 °C with an interval of 5 °C, and 200 rpm for 4 days. Timing sampling was done during the cultivation. The bacterial density in each sample was measured at a wavelength of 600 nm (OD_600_) to draw a growth curve. Each sample was centrifuged at 12,000× *g*, 4 °C for 12 min, and the supernatant was collected to measure the alginate lyase activity at its optimal temperature. The optimal temperature for alginate lyase activity was determined by measuring the enzyme activity from 10 °C to 60 °C with an interval of 10 °C. 

## 3. Results

### 3.1. Diversity of Bacteria Associated with the Arctic Brown Alga *Laminaria*

**Figure 1:**
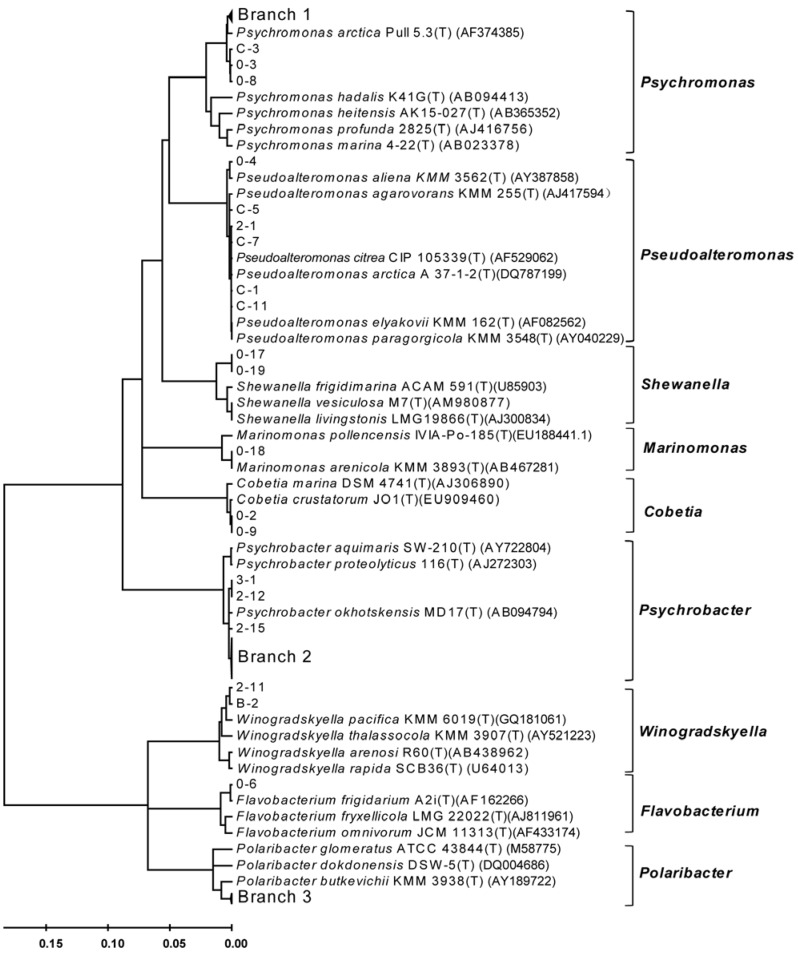
A neighbor-joining phylogenetic tree of the bacteria recovered from the six *Laminaria* samples on the Arctic Ocean based on the 16S rRNA gene sequences. Branch 1 includes *Psychromonas* strains 0-7, 3-5, 3-6, 3-12, 3-14, C-2 and C-8. Branch 2 includes *Psychrobacter* strains 0-5, 0-10, 0-12, 0-13, 0-14, 0-15, 0-20, 1-1, 1-2, 1-3, 1-4, 1-5, 2-3, 2-4, 2-7, 2-10, 2-13, 2-14, 3-2, 3-3, 3-4, 3-7, 3-8, 3-9, 3-10, 3-11, 3-13, 3-15, 3-16 and 3-17, and *Psychrobacter fozii* NF23(T) (AJ430827). Branch 3 includes *Polaribacter* strains 0-11, 2-2, 2-5, 2-6, 2-8, 2-9, 2-16 and 2-17.

After cultivation of the bacteria enriched from the Arctic *Laminaria* on screening plates, a lot of colonies appeared on the plates with 10^−^^2^–10^−^^5^ diluted samples. More than 70 isolates were purified and subjected to 16S rRNA gene amplification. Isolates with the same (or only one base difference) 16S rRNA gene sequence were considered as the same strain. According to an alignment of the 16S rRNA gene sequences, a total of 65 different strains were isolated from the Arctic *Laminaria* samples. A phylogenetic analysis based on the 16S rRNA gene sequences showed that the recovered *Laminaria*-associated bacteria belonged to nine genera, namely *Cobetia*, *Psychrobacter*, *Winogradskyella*, *Psedoalteromonas*, *Psychromonas*, *Polaribacter*, *Shewanella*, *Flavobacterium* and *marinomonas* (Figure 1). *Psychrobacter* (33/65) were the most predominant group. *Psychromonas* (10/65) and *Polaribacter *(8/65) also showed some preponderance. 

### 3.2. Diversity of Alginate Lyase-Excreting Bacteria from the Arctic Brown Alga *Laminaria*

**Figure 2:**
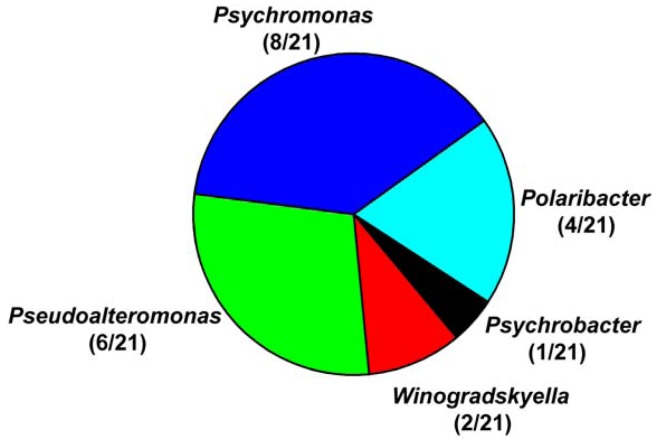
Abundances of the phylotypic groups of cultivable alginate lyase-excreting bacteria recovered from the brown alga *Laminaria* from six sample stations on the Arctic Ocean.

Alginate lyase-excreting bacteria were further screened from the recovered *Laminaria*-associated isolates by screening plates. Twenty one strains showed clear haloes after 10% cetylpyridiniun chloride was flooded on the plates (Supplementary Materials, Figure S1), indicating that these strains could excrete alginate lyase. The 21 strains were taken as alginate lyase-excreting bacteria for further study. These strains belonged to five genera, namely *Psychrobacter*, *Winogradskyella*, *Psedoalteromonas*, *Psychromonas* and *Polaribacter*. *Psychromonas* (8/21), *Psedoalteromonas* (6/21) and *Polaribacter* (4/21) were the predominant genera (Figure 2). Therefore, it seemed that the predominant genera of the *Laminaria*-associated bacteria and the alginate lyase-excreting bacteria showed some difference.

**Figure 3:**
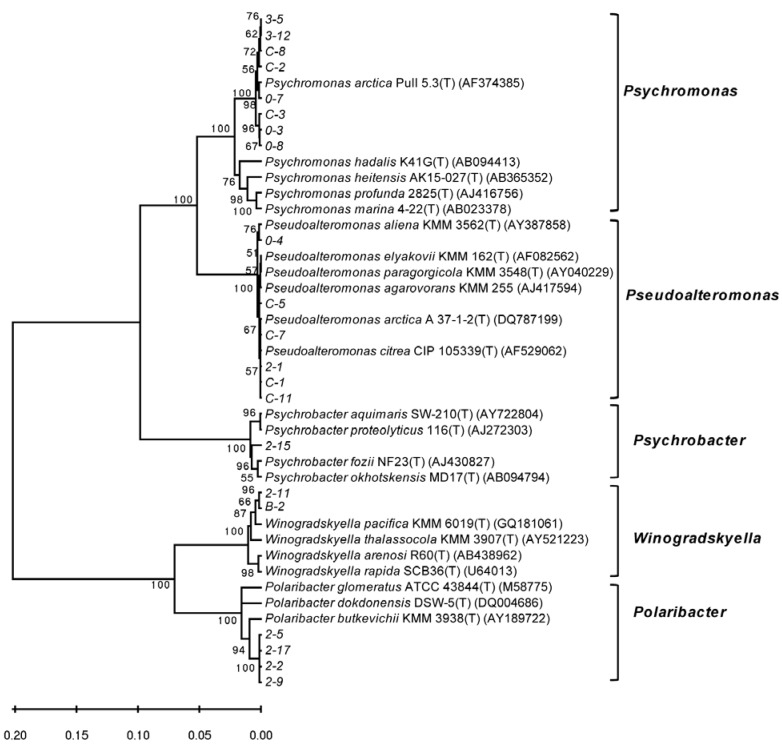
A neighbor-joining phylogenetic tree of the alginate lyase-excreting bacteria recovered from the Arctic brown alga *Laminaria* based on the 16S rDNA sequences.

A distance-based neighbor-joining tree was constructed with the 16S rRNA gene sequences of the alginate lyase-excreting bacteria in this study and reference sequences from the GenBank database (Figure 3). There were three predominant branches, namely *Psychromonas*, *Pseudoalteromonas* and *Polaribacter*. Strains related to *Psychromonas* were the most frequently recovered (recovered from three *Laminaria* samples) and formed the largest group in terms of abundance (8 of 21 strains, Figure 3). These *Psychromonas* strains showed high similarity (more than 98.3%) with *Psychromonas arctica* Pull 5.3^T^ that was isolated from a seawater sample collected from Spitzbergen in the Arctic (Supplementary Materials, Table S1). Four strains, 2-2, 2-5, 2-9 and 2-17, showed high similarity (more than 98.5%) with *Polaribacter butkevichii* KMM 3938^T^ that was isolated from the seawater collected from the Sea of Japan, Russia. Strain 2-15 exhibited a distant relationship with any of the previously identified species. It may represent a potentially new species, which merits further study.

### 3.3. Optimal Temperatures for Growth and Alginate Lyase Production of the Strains

Among the 21 alginate lyase-excreting strains, 20 strains had the optimal temperatures for growth at 10–25 °C, indicating that most alginate lyase-excreting strains are cold-adapted bacteria (Table 2). The optimal growth temperature was just 10 °C for strains 0-7 and 2-1, and 15 °C for strains 0-4, 2-17 and 3-12, showing that these five strains are psychrophiles. The other stains with an optimal growth temperature of 20 °C, 25 °C or 30 °C are psychrotrophiles. Correspondingly, the optimal temperatures for the strains to produce alginate lyases were also low. Ten strains showed the same optimal temperature for growth and alginate lyase production. However, the optimal temperature for some strains to produce extracellular alginate lyases was lower than that for growth, such as strains 0-8, 2-11 and 3-12, while others showed higher optimal temperatures for alginate lyase production than for growth. In general, the strains showed low optimal temperatures for growth and extracellular enzyme production, reflecting their adaptation to the cold Arctic Ocean. 

**Table 2:** Optimal temperatures for growth and alginate lyase production of the strains and for the activity of the alginate lyase from each strain.

Genera	Strains	Optimum Temperature( °C)		
		for Growth	for Enzyme Production	for Enzyme Activity
*Polaribacter*	2-2	20	25	30
	2-9	20	25	50
	2-5	25	25	50
	2-17	15	25	40
*Pseudoalteromonas*	0-4	15	15	30
	2-1	10	25	40
	C-1	30	25	20
	C-5	25	25	40
	C-7	25	30	30
	C-11	25	25	40
*Psychrobacter*	2-15	20	20	50
*Psychromonas*	0-3	20	20	30
	0-7	10	20	45
	0-8	20	15	30
	3-5	20	20	30
	3-12	15	10	30
	C-2	20	20	20
	C-3	20	25	20
	C-8	25	25	30
*Winogradskyella*	2-11	20	15	40
	B-2	25	25	40

### 3.4. Optimal Temperatures for the Enzymatic Activity of the Alginate Lyases from the Strains

The optimal temperatures for the extracellular alginate lyases produced by the 21 strains were ranged from 20 °C to 50 °C (Table 2). The alginate lyases produced by 11 strains showed the highest activity at 20–30 °C, indicating the good cold-adaptation of these alginate lyases. The alginate lyases produced by the other 10 strains showed the highest activity at 40–50 °C. The differences in the optimal temperatures for enzyme activity suggest that the alginate lyases excreted by these strains may be different kinds of alginate lyases. The results indicate that a majority of bacterial extracellular alginate lyases from the Arctic Ocean are cold-adapted enzymes.

### 3.5. Levels of Extracellular Alginate Lyase Activity of the Strains

**Figure 4:**
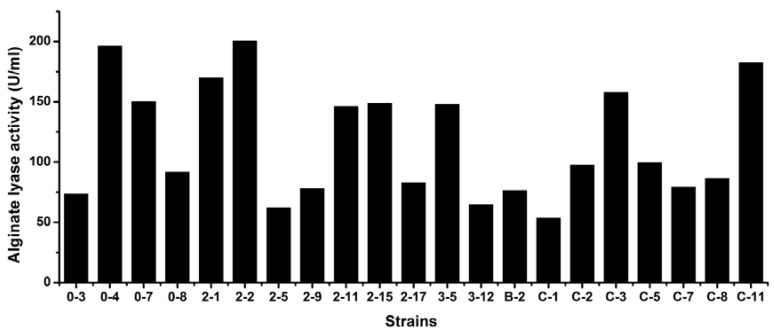
Extracellular alginate lyase activity levels of the strains. Each strain was cultured at its optimal temperature, and the alginate lyase activity at its optimal temperature for each strain was determined.

The extracellular alginate lyase activity level of every strain was determined by measuring the alginate lyase activity in the culture at its optimal temperature (Figure 4). Among the 21 alginate lyase-excreting strains, strain 2-2 had the highest extracellular alginate lyase activity level, more than 200 U/mL, while strain C-1 had the lowest extracellular alginate lyase activity level, lower than 60 U/mL. Strains 0-4 and C-11 also had a level of near 200 U/mL. This indicated that these strains may be good candidates for isolation of novel alginate lyases. 

## 4. Discussion

Alginate lyases from bacterial sources have been used in analyzing alginate structure, protoplasting of seaweed, studying *Fucus* cell wall development, and producing polyM, or polyG blocks [16,17,18,19,20]. Recently alginate oligomers with the specific molecular weight have been shown to have specific bio-activities, such as stimulating the secretion of cytotoxic cytokines from human macrophage [21] or promoting the growth of bifidobacteria [22]. Alginate lyases can be used as tools for generating bio-active products. The use of alginate lyase for the treatment of cystic fibrosis suffers still remains one of the most important goals of studying alginate lyase [23]. Nowadays fossil fuel resources are running out and this has ignited efforts to produce bio-fuels and renewable commodity chemical compounds via microbial fermentation. An engineered microbial platform with a bifunctional alginate lyase aly-SJ02 was generated, which can simultaneously degrade, uptake and metabolize alginate [24]. Discovery of new alginate lyase will facilitate the application of alginate lyase in the production of biofuel and bioactive alginate hydrolysates.

Diversities of alga-associated bacteria and algiante lyase-excreting bacteria in the Arctic Ocean have not been reported. In this study, the diversity of alga-associated bacteria from 6 *Laminaria* samples was investigted, which showed that *Psychrobacter*, *Psychromonas* and *Polaribacter* were the most predominant groups of the 65 recovered *Laminaria*-associated bacteria. To dig the algiante lyase resource in the Arctic Ocean, algiante lyase-excreting bacteria were further screened from the 65 *Laminaria*-associated bacteria. Alginate lyase-excreting marine bacteria so far reported include *Alginovibrio*, *Alteromonas*, *Beneckea*, *Halomonas*, *Photobacterium*, *Pseudoalteromonas* and *Vibro* [9]. In this study, 21 algiante lyase-excreting strains were recovered from the Arctic brown alga *Laminaria*. Besides *Pseudoalteromonas*, it was first found that *Psychrobacter*, *Winogradskyella*, *Psychromonas* and *Polaribacter* excrete alginate lyases. Among these algiante lyase-excreting strains, the optimal temperatures for growth and algiante lyase production of many strains were as low as 10–20 °C, showing that they are psychrophilic bacteria. And the alginate lyases excreted by 11 strains showed the highest activity at 20–30 °C, indicating that these alginate lyases are cold-adapted enzymes. Moreover, some strains showed high extracellular alginate lyase activity levels around 200 U/mL. These results suggest that the algiante lyase-excreting bacteria we recovered from the Arctic alga *Laminaria* may be good materials for studying bacterial cold-adapted alginate lyases. Cold-adapted alginate lyases can offer advantages over the currently used alginate lyases in the industrial processes where heating is economically counterproductive or where low temperatures are required. In addition, there can also be considerable energy savings for those reactions that can be efficiently conducted at lower temperatures. Cold-adapted enzymes usually have low thermal stability and can be easily inactivated at moderate temperatures, which have advantages in the industrial processes that do not permit high temperatures for enzyme inactivation.

## Supplementary Files

Supplementary File 1: PDF-Document (PDF, 103 KB)
