# Supplementary material for: Cultivable Alginate Lyase-Excreting Bacteria Associated with the Arctic Brown Alga Laminaria
Source: Mar Drugs. 2012 Nov 6;10(11):2481–91. doi: 10.3390/md10112481 (PMC3509530; doi:10.3390/md10112481)
Supplement: Supplementary File 1: — PDF-Document (PDF, 103 KB) [file marinedrugs-10-02481-s001.pdf]

Supplementary Materials

Figure S1. The clear zone of the isolates formed on the screening plates after flooding with 10% (wt/vol) calcium chloride.

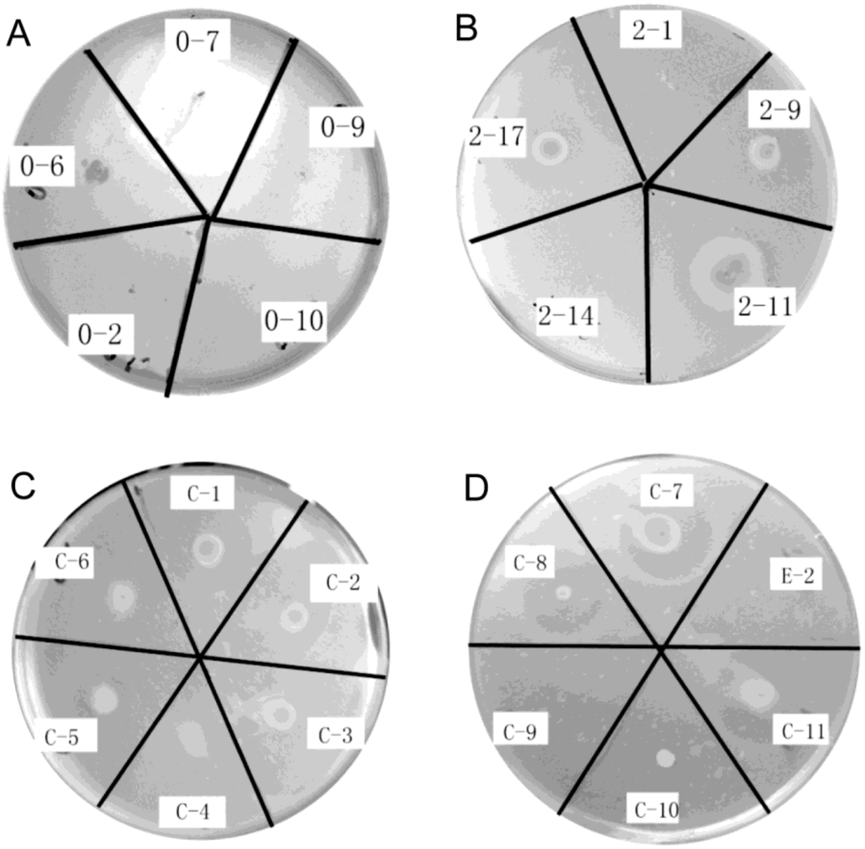

Table S1. Species affiliation of the 21 isolates.

	**Name/Title**	**Strain**	**Accession**	**Pairwise Similarity**	**Diff/Total nt**
0-3	*Psychromonas arctica*	Pull 5.3(T)	AF374385	98.535	22/1502
0-4	*Alteromonas fuliginea*	CIP 105339(T)	AF529062	99.373	7/1117
0-7	*Psychromonas arctica*	Pull 5.3(T)	AF374385	99.002	15/1503
0-8	*Psychromonas arctica*	Pull 5.3(T)	AF374385	98.337	25/1503
2-1	*Alteromonas fuliginea*	CIP 105339(T)	AF529062	99.932	1/1472
2-2	*Polaribacter butkevichii*	KMM 3938(T)	AY189722	98.563	20/1392
2-3	*Psychrobacter fozii*	NF23(T)	AJ430827	99.865	2/1477
2-5	*Polaribacter butkevichii*	KMM 3938(T)	AY189722	98.702	18/1397
2-9	*Polaribacter butkevichii*	KMM 3938(T)	AY189722	98.532	21/1431
2-11	*Winogradskyella pacifica*	KMM 6019(T)	GQ181061	98.241	25/1421
2-15	*Psychrobacter fozii*	NF23(T)	AJ430827	97.497	18/1385
2-17	*Polaribacter butkevichii*	KMM 3938(T)	AY189722	98.742	36/1438
3-5	*Psychromonas arctica*	Pull 5.3(T)	AF374385	98.47	23/1503
3-12	*Psychromonas arctica*	Pull 5.3(T)	AF374385	98.47	23/1504
C-1	*Pseudoalteromonas arctica*	A 37-1-2(T)	DQ787199	99.799	3/1494
C-2	*Psychromonas arctica*	Pull 5.3(T)	AF374385	98.736	19/1503
C-3	*Psychromonas arctica*	Pull 5.3(T)	AF374385	98.337	25/1503
C-5	*Pseudoalteromonas arctica*	A 37-1-2(T)	DQ787199	99.197	12/1494
C-7	*Pseudoalteromonas arctica*	A 37-1-2(T)	DQ787199	100	0/1494
C-8	*Psychromonas arctica*	Pull 5.3(T)	AF374385	98.669	20/1503
C-11	*Pseudoalteromonas arctica*	A 37-1-2(T)	DQ787199	99.799	3/1494
B-2	*Winogradskyella pacifica*	KMM 6019(T)	GQ181061	98.67	19/1429
